# Development of a novel combined nomogram model integrating deep learning-pathomics, radiomics and immunoscore to predict postoperative outcome of colorectal cancer lung metastasis patients

**DOI:** 10.1186/s13045-022-01225-3

**Published:** 2022-01-24

**Authors:** Renjie Wang, Weixing Dai, Jing Gong, Mingzhu Huang, Tingdan Hu, Hang Li, Kailin Lin, Cong Tan, Hong Hu, Tong Tong, Guoxiang Cai

**Affiliations:** 1 Department of Colorectal Surgery, Fudan University Shanghai Cancer Center, 270 Dong’an Road, Shanghai, 200032 China; 2 Department of Oncology, Shanghai Medical College, Fudan University, Shanghai, China; 3 Department of Radiology, Fudan University Shanghai Cancer Center, 270 Dong’an Road, Shanghai, 200032 China; 4 Department of Medical Oncology, Fudan University Shanghai Cancer Center, Shanghai, China; 5 Department of Thoracic Surgery, Fudan University Shanghai Cancer Center, 270 Dong’an Road, Shanghai, 200032 China; 6 Department of Pathology, Fudan University Shanghai Cancer Center, Shanghai, China

**Keywords:** Colorectal cancer, Lung metastasis, Pathomics, Radiomics, Immunoscore, Nomogram

## Abstract

Limited previous studies focused on the death and progression risk stratification of colorectal cancer (CRC) lung metastasis patients. The aim of this study is to construct a nomogram model combing machine learning-pathomics, radiomics features, Immunoscore and clinical factors to predict the postoperative outcome of CRC patients with lung metastasis. In this study, a total of 103 CRC patients having metastases limited to lung and undergoing radical lung resection were identified. Patch-level convolutional neural network training in weakly supervised manner was used to perform whole slides histopathological images survival analysis. Synthetic minority oversampling technique and support vector machine classifier were used to identify radiomics features and build predictive signature. The Immunoscore for each patient was calculated from the density of CD3+ and CD8+ cells at the invasive margin and the center of metastatic tumor which were assessed on consecutive sections of automated digital pathology. Finally, pathomics and radiomics signatures were successfully developed to predict the overall survival (OS) and disease free survival (DFS) of patients. The predicted pathomics and radiomics scores are negatively correlated with Immunoscore and they are three independent prognostic factors for OS and DFS prediction. The combined nomogram showed outstanding performance in predicting OS (AUC = 0.860) and DFS (AUC = 0.875). The calibration curve and decision curve analysis demonstrated the considerable clinical usefulness of the combined nomogram. Taken together, the developed nomogram model consisting of machine learning-pathomics signature, radiomics signature, Immunoscore and clinical features could be reliable in predicting postoperative OS and DFS of colorectal lung metastasis patients.

**Supplementary Information:**

The online version contains supplementary material available at 10.1186/s13045-022-01225-3.

**To the editor**,

Though great efforts have been made on the prognosis prediction of colorectal cancer liver metastases, literatures on colorectal cancer (CRC) patients with lung metastasis are limited [1, 2]. Unlike other distant metastases (liver, peritoneum, etc.), lung metastases grow relatively slow and have superior prognosis [3]. Therefore, treatment mode of metastases from other sites cannot be fully referred and accurate prediction of long-term death and progression risk for lung metastases of CRC patients remains challenging. More prognostic factors should be exploited to facilitate the risk stratification for these patients.

Recently, the computational analysis of medical images in particular, pathomics and radiomics has shown much exciting results for the prediction of the prognosis of CRC patients [4, 5]. More interestingly, preliminary results from radiomics and pathomics analysis have demonstrated their ability to correlate image features with the status of in-situ immune cell infiltrate which is newly identified exceptional biomarker for prognosis and immunotherapy benefit prediction [6–9]. To date, the application of radiomics, pathomics and immunoscore based on metastatic sites in CRC patients with lung metastasis for overall survival (OS) and disease free survival (DFS) prediction has not been reported. We proposed that development of a combined model based on the pathomics, radiomics and immunoscore may provide a reliable estimate of the risk of recurrence and death in patients with lung metastasis. In this study, a total of 103 CRC patients with metastases limited to lung were identified from Fudan University Shanghai Cancer Center. The clinical and histopathological characteristics of the patients are shown in Additional file 3: Table S1.

As previously reported [10] (Additional file 1: Methods), the whole slide histopathological images survival analysis framework (WSISA) based on conventional haematoxylin and eosin stained images was used to develop the pathomics signature. Five whole slide features were selected for the construction of pathomics-based prognosis model, which could automatically distinguish patients with worse survival outcomes, with hazard ratio value of 8.09 for OS prediction (95% confidence interval: 3.38–19.38, *p* = 0.0001, Additional file 2: Fig. S1a) and 2.51 for DFS prediction (95% confidence interval: 1.31–4.80, *p* = 0.005, Additional file 2: Fig. S1b).

By applying synthetic minority oversampling technique and support vector machine classifier (Additional file 1: Methods), eight features were finally identified to develop the radiomics signatures. The detailed procedure of features selection and radiomics signature development were described in Additional file 1: Methods. Additional file 2: Fig. S2(a) and (b) showed the features selected in DFS and OS prediction model, respectively. The high and low radiomics score subtypes were determined by operating the threshold of 50% to prediction scores of two radiomics models. It showed that the developed two radiomics signatures can divide patients into low and high risk radiomic subtypes with significantly different DFS (Additional file 2: Fig. S3, *p* = 0.0001) and OS (Additional file 2: Fig. S3, *p* = 0.0006).

Based on established procedure (Additional file 1: Methods), we calculated Immunoscore for each patients and the distribution of patients with high tumor density of CD3 and CD8 (≥ 75th percentile) in different regions was shown in Additional file 3: Table S2. A total of 21 (20.4%) patients were categorized into high immune score group (Additional file 2: Fig. S4). Further survival analysis showed that patients with high immune score have notably improved DFS and OS, confirming immunoscore based on lung metastatic site as a good prognostic factor in CRC patients with lung metastasis (Additional file 2: Fig. S5). To test the correlation between pathomics/radiomics signatures and immunoscore, we compared the difference of pathomics/radiomics score between low and high immune score groups. It was found that patients with high immune score have significantly lower pathomics/radiomics score (Additional file 2: Fig. S6a–d). Further, we characterized the distribution of pathomics/radiomics scores and immune status and it suggested that patients with low pathomics/radiomics scores generally had high immune score than that of those with higher radiomics scores (Additional file 2: Fig. S6e–h), confirming the intimate association between pathomics/radiomics and immune infiltrating status.

After multivariate analysis adjusting by clinicopathological variables, the pathomics, radiomics signatures and immunoscore remained powerful and independent factors in predicting OS and DFS (Additional file 3: Table S3). Based on the multivariate analysis, two nomograms, integrating the pathomics, radiomics signature, immunoscore and clinical risk factors, were formulated to predict the OS and DFS respectively (Fig. 1b, c). For the OS prediction, sex, immunoscore, pathomics and radiomics signature were integrated into the death predicting nomogram. For DFS prediction, tumor site is another independent prognostic factor. The usefulness of combined nomogram was also confirmed in the survival ROC analysis with an area under curve (AUC) of 0.860 for predicting OS (Fig. 2a) and an AUC of 0.875 for predicting DFS (Fig. 2b). The AUC values revealed the high performance of prognosis prediction using the combined nomograms. Further, the calibration curve showed a high accuracy of the combined nomogram model for predicting OS (Fig. 2c) and DFS (Fig. 2d). The decision curve analysis was then performed to illustrate the clinical decision utility of the combined nomogram. When predicting OS (Fig. 2e) and DFS (Fig. 2f), the combined model showed a higher area under the decision curve than that using the pathomics signature, radiomics signature and immunoscore alone.

**Fig. 1:**
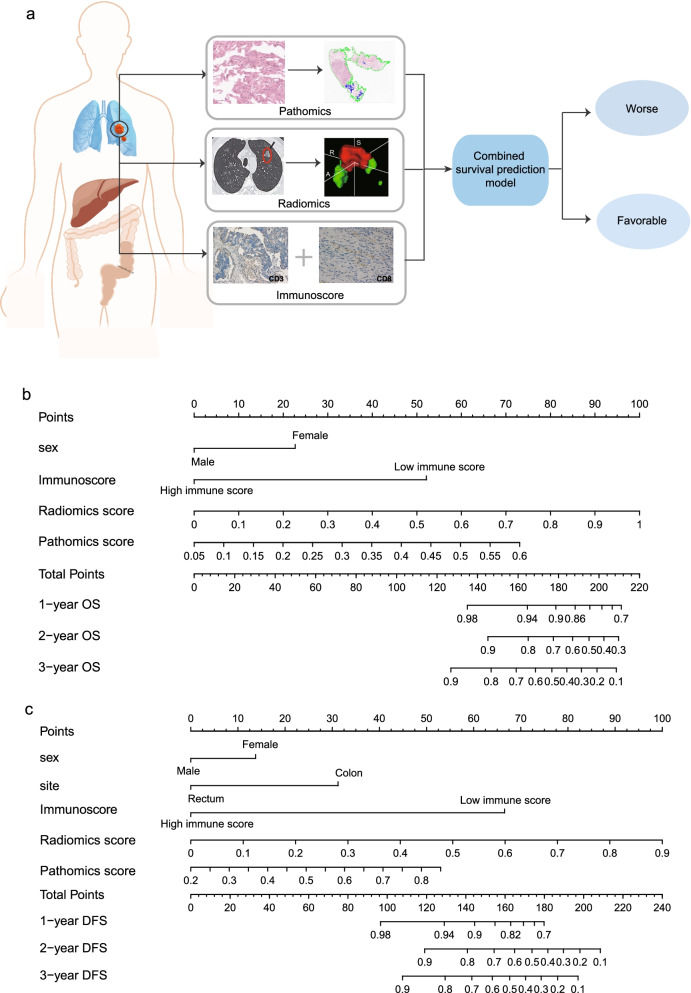
(**a**) Schematic of the combined predictive model. Combined nomograms incorporating clinical features, pathomics, radiomics signature and Immunoscore for OS (**b**) and DFS (**c**) prediction

**Fig. 2:**
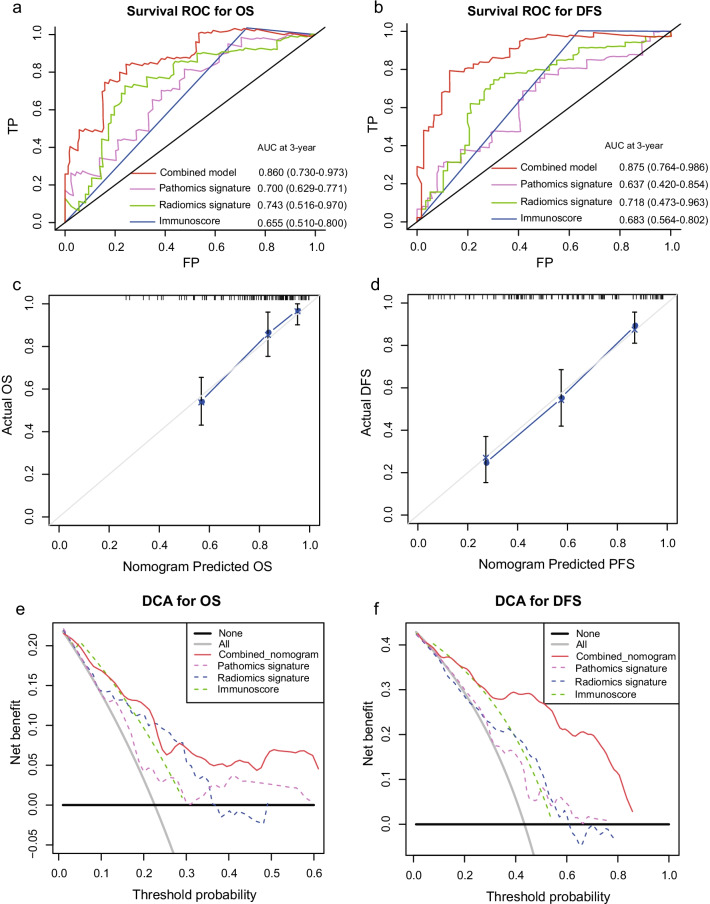
Survival ROC curves demonstrate the prognostic accuracy in predicting OS (**a**) and DFS (**b**) of the combined nomograms. Calibration curves of combined nomograms for OS (**c**) and DFS (**d**) prediction. Decision curve analysis demonstrate the clinical utility in predicting OS (**e**) and DFS (**f**) of the combined nomograms, radiomics signature and Immunoscore

In summary, the combined nomogram model developed in this study can effectively predict the OS and DFS for CRC patients with lung metastasis. This prediction tool may help to identify high risk patients who require more aggressive therapeutic intervention and follow-up scheme.

## Supplementary Information

**Additional file 1**. **Supplementary Methods**.

**Additional file 2** . **Supplementary Figures**. **Figure S1**. Kaplan-Meier survival curves depicting OS (a) and DFS (b) according to pathomics subtypes. Significant difference in survival of patients was observed between high and low pathomics score. **Figure S2**. Boxplots of the selected imaging features in radiomics model. (a) shows the features selected in DFS prediction model, (b) shows the features selected in OS prediction mode. **Figure S3**. Kaplan–Meier survival curve of 103 CRC patients with lung metastasis for estimating DFS (a) and OS (b) according to radiomic subtypes. The low and high radiomic score are the patients with prediction scores ≤50% and >50% respectively. **Figure S4**. Percentile distribution of Immunoscore in CRC patients with lung metastasis. **Figure S5**. Kaplan-Meier survival curves depicting OS (a) and DFS (b) according to Immunoscore. Significant difference in survival of patients was observed between high and low immune score. **Figure S6**. Comparison of pathomics/radiomics score of OS (a, c) and DFS (b, d) prediction model between patients in low and high immune score group. Distribution of pathomics/radiomics score of OS (d, f) and DFS (e, g) prediction model and immune status.

**Additional file 3** . **Supplementary Tables**. **Table S1**. Clinicopathological characteristics of colorectal lung metastasis patients. **Table S2**. Distribution of patients with high CD3/CD8 cell density (≥75th percentile) in different regions. **Table S3**. Multivariate Cox analysis of OS and PFS.

## Data Availability

The datasets used and/or analyzed during the current study are available from the corresponding author on reasonable request.

## Abbreviations

CRC: Colorectal cancer
OS: Overall survival
DFS: Disease free survival
AUC: Area under curve
WSISA: Whole slide histopathological images survival analysis framework
