# Supplementary material for: Development of a novel combined nomogram model integrating deep learning-pathomics, radiomics and immunoscore to predict postoperative outcome of colorectal cancer lung metastasis patients
Source: J Hematol Oncol. 2022 Jan 24;15:11. doi: 10.1186/s13045-022-01225-3 (PMC8785554; doi:10.1186/s13045-022-01225-3)
Supplement: Supplementary file 1 — Additional file 1. Supplementary Methods. [file 13045_2022_1225_MOESM1_ESM.docx]

**Additional file 1: Methods**

*Study population*

The data of all patients who underwent lung metastasectomy for CRC between 2007 to 2017 in Fudan University Shanghai Cancer Center (FUSCC) were retrospectively analyzed. Written informed consent was obtained from all study participants, and study protocol was approved by the Ethics Committee of FUSCC. Preoperative evaluation included clinical examination, blood test, cardiological evaluation, chest and abdomen CT scan and lung function examination. All patients were discussed by our Multidisciplinary team (MDT) and suggested that radical lung metastasectomy was the best treatment. Sex, age, CRC staging (TNM, AJCC8th edition), tumor site/size/shape/differentiation/neural invasion/lymphovascular invasion of primary CRC, CEA before primary surgery and lung metastatic surgery were collected. Type of lung surgery (wedge resection, segmentectomy, lobectomy, bilobectomy or pneumonectomy), approach (VATS or thoracotomy) and lymphadenectomy were performed according to surgeon’s decision. Radical resection (R0 resection, which was defined by pathologists as negative surgical margins macro and microscopically) was achieved in all patients. All specimens were reviewed by 2 experienced pathologists and immunohistochemistry (CK20, CDX2, TTF-1) was done for distinguishing lung metastases and primary lung cancer. The inclusion criteria include:(1) had lung metastases confirmed by pathologists; (2) underwent radical pneumectomy; (3) had complete medical records including demographic, clinical and pathological data; (4) had at least 6 months follow-up time after surgery. Patients who (1) had metastases outside the lung; (2) lost follow-up or died within 6 months; (3) did not sign the informed consent were all excluded from the study. The follow-up was obtained from internal institutional database or by phone-call.

*Machine learning-pathomics signature construction*

As previously reported[1], the Whole Slide Histopathological Images Survival Analysis framework (WSISA) based on conventional haematoxylin and eosin stained images was used to develop the pathomics signature. Briefly, this procedure consists of four main stages (Figure 1):

1) generating candidate patches from the Whole Slide Histopathological Images (WSIs);

A fixed area sampling ratio was set to sample the candidate patches from WSIs (patch size of 512 by 512, 0.5 microns per pixel)

2) performing phenotypes based clustering of patch candidates;

To distinguish the patches from different sections (tumor/normal/both), clustering was performed based on their phenotypes. Considering the high dimension of generated features, PCA was used to reduce the dimension before the implement of K-means clustering process.

3) identifying targeted clusters according to performance of patch-wise survival prediction;

Patch-wise trained DeepConvSurv models were performed and the clusters with predicting accuracy a little bit better than random guess were selected.

4) aggregating the finally identified clusters to make survival prediction.

Based on specific calculation formula, patient-wise survival prediction values were calculated based on two consecutive steps of generating weighted features and
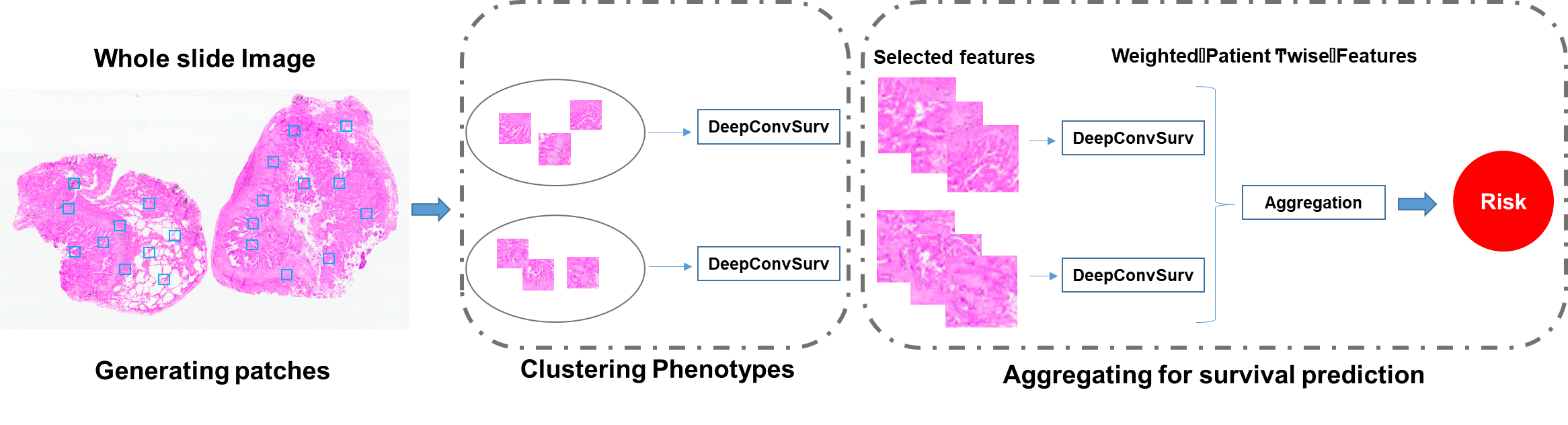
aggregation.

Figure 1. An overview of established WSISA framework.

*Radiomics model development*

To predict the survival or prognosis statuses of CRC patients, we developed a CT image based radiomics model by using applying machine learning classifier. A radiologist (Tingdan Hu) with 5-year medical imaging interpretation experience delineated the boundary of tumor in CT images. All 3D tumors weges by using the ITK-Snap software (<http://www.itksnap.org>). For case with multiple tumors, the metastatic one was selected by reviewing the pathological report. In order to reduce the biases of image resolution, we used a B-spline interpolation technique to standardize the CT images. Then, all the voxel spacing of all CT scan were normalized to 1mm×1mm×1mm.

To decode the imaging phenotypes of lung metastasis, we extracted 1106 radiomics features to quantify each segmented tumor[1]. These features were computed by using three types of image namely, original CT image, LoG image and wavelet image, respectively. LoG image was generated by filtering original CT image with Laplacian of Gaussian (LoG) filter. The σ of LoG filter was 1, 2 and 3. Wavelet image was generated by filtering original CT image with wavelet filter. Eight decompositions wavelet image per level were yielded by applying either a high or a low pass filter in each of the three dimensions. The wavelet function of wavelet filter was “coif1”. After performing filtering process, we calculated histogram feature, shape feature and texture feature by using three types of images. Among the 1106 radiomics features, 105 were original image features, 273 were LoG features, and 728 were wavelet features. The original image features involved 18 histogram features, 14 shape features and 73 texture features. The texture features were consisted of 22 gray level co-occurrence matrix (GLCM) features, 14 gray level dependence matrix (GLDM) features, 16 gray level run length matrix (GLRLM) features, 16 gray level size zone matrix (GLSZM) features, and 5 neighboring gray tone difference matrix (NGTDM) features. After extracting radiomics features, we normalized the value of each feature to [0, 1].

Then, we applied a recursive feature elimination (RFE) method configured with L1-based feature selection estimator to select optimal imaging features from initial radiomics features. To build the feature selection estimator, we used a Lasso model with a α value of 0.005. By using the feature selection technique, we reduced the dimensionality of radiomics features, and built an optimal feature pool to train/test a classification model. Due to unbalanced dataset, we used a synthetic minority oversampling technique (SMOTE) to resample the cases in our dataset [2]. Then, we applied a support vector machine (SVM) classifier to build the classification model to predict the survival status of patients. During this process, we used a leave-one-out cross-validation (LOOCV) method to train and test the SVM classifier. In each training and testing process cycle, we selected one case to test the model performance and used the other samples to train the classifier. To avoid the biases during dataset partition, we embedded SMOTE and SVM classifier into LOOCV process. In the LOOCV training/testing process, we only used the real sample in our dataset to validate the model performance, and obtained 103 prediction scores of 103 cases in our dataset. By analyzing the prediction scores of the metastatic patients, we applied a default threshold

of 50% for prediction probability to divide the patients into high-risk and low-risk group.

Figure 2. The flowchart of the proposed radiomics model

*Calculation of Immunoscore*

The whole slide of specimens was used to calculate the CD3/CD8 Immunoscore (I)[2]. CD3- and CD8-stained slides were scanned at 40 × magnification using Leica SCN400 slide scanner (Leica Microsystems, Wetzlar, Germany) and uploaded to image analysis software, Halo (Indica Labs) to determine the densities of CD3+ and CD8+ T cells in colon tumor and invasive margin regions. Cases with mean density ≥ 75-percentile were regarded as “high” density[3]. Briefly, Immunoscore ranging from 0 (I0), when low densities of two cell types are found in both regions, to 4 (I4), when high densities are found of two cell types in both regions. Consistent with previous studies, patients with I0-I2 were classified into low immune score group, while patients with I3-I4 were classified into high immune score group

*Development of a combined nomogram with radiomics, Immunoscore and clinical, features*

To identify independent prognostic factors, variables achieving a significance of P < 0.05 were selected for multivariable analyses via the Cox regression model. On the basis of the results of the multivariable analysis, two nomograms, integrating the radiomics signature, Immunoscore and other clinicopathological risk factors, was formulated to predict the OS and PFS for CRC patients with lung metastasis.

Nomograms have been successfully applied to a variety of malignancies for the purpose of improving oncological outcome prediction and provide patients and physicians with a more intelligible outcome measure when making treatment-related decisions. Upon using nomogram, it can be interpreted by summing up the weighted score assigned to each variable, which is indicated at the top of scale. The total score can be converted to predicted probability of death and recurrence or metastasis for a patient in the lowest scale. A higher total points was associated with a worse OS and DFS.

*Survival analysis and performance evaluation*

To assess the association between the high-risk or low-risk prediction scores generated by radiomics model and patients’ PFS and OS, we used several statistical analysis methods, which included Survival ROC analysis, Kaplan-Meier (KM) survival analysis, and Cox proportional risk regression model. In this process, we first evaluated the prediction performance of our classification model by using C-index estimator. Then, we estimated cumulative survival rate by using KM survival analysis method. Finally, we used Cox proportional risk regression to estimate the hazard ratio (HR) with 95% confidence interval (CI). Survival decision curve analysis (DCA) was further used to evaluate the net benefit derived from the two signatures. The clinical utility could be demonstrated by quantifying the net benefits of a series of threshold probabilities in the queue. Decision curve analysis examined the theoretical relationship between the threshold death or relapse probability of patients and the relative value of false-positive and false-negative results to determine the predictive ability of the prediction model. Variables with statistically significant p values in univariate analysis were included in multivariate analysis. All tests were 2-sided, and P < 0.05 was considered statistically significant.

In this study, we implemented the radiomics model process by using the Python programming. And we performed statistical analyses with R software, version 3.5.3 (F Foundation for Statistical Computing, Vienna, Austria). All these steps were implemented on a computer configured with Intel Core i7-8700 CPU 3.2GHz × 2 and 16 GB RAM.

1 Zhu X, Yao J, Zhu F,Huang J. WSISA: making survival prediction from whole slide histopathological images. In: 2017 IEEE Conference on Computer Vision and Pattern Recognition (CVPR), IEEE Computer Society, Los Alamitas, CA, 7234–7242.

2 Fridman W H, Pagès F, Sautès-Fridman C,Galon J. The immune contexture in human tumours: impact on clinical outcome. Nat Rev Cancer, 2012, 12(4): 298-306.

3 Lea D, Watson M, Skaland I, Hagland H R, Lillesand M, Gudlaugsson E, et al. A template to quantify the location and density of CD3 + and CD8 + tumor-infiltrating lymphocytes in colon cancer by digital pathology on whole slides for an objective, standardized immune score assessment. Cancer Immunol Immunother, 2021, 70(7): 2049-2057.
