# Supplementary material for: Development of a novel combined nomogram model integrating deep learning-pathomics, radiomics and immunoscore to predict postoperative outcome of colorectal cancer lung metastasis patients
Source: J Hematol Oncol. 2022 Jan 24;15:11. doi: 10.1186/s13045-022-01225-3 (PMC8785554; doi:10.1186/s13045-022-01225-3)
Supplement: Supplementary file 2 — Additional file 2 . Supplementary Figures. Figure S1. Kaplan-Meier survival curves depicting OS (a) and DFS (b) according to pathomics subtypes. Significant difference in survival of patients was observed between high and low pathomics score. Figure S2. Boxplots of the selected imaging features in radiomics model. (a) shows the features selected in DFS prediction model, (b) shows the features selected in OS prediction mode. Figure S3. Kaplan–Meier survival curve of 103 CRC patients with lung metastasis for estimating DFS (a) and OS (b) according to radiomic subtypes. The low and high radiomic score are the patients with prediction scores ≤50% and >50% respectively. Figure S4. Percentile distribution of Immunoscore in CRC patients with lung metastasis. Figure S5. Kaplan-Meier survival curves depicting OS (a) and DFS (b) according to Immunoscore. Significant difference in survival of patients was observed between high and low immune score. Figure S6. Comparison of pathomics/radiomics score of OS (a, c) and DFS (b, d) prediction model between patients in low and high immune score group. Distribution of pathomics/radiomics score of OS (d, f) and DFS (e, g) prediction model and immune status. [file 13045_2022_1225_MOESM2_ESM.docx]

**Additional file 2: Figures**

**
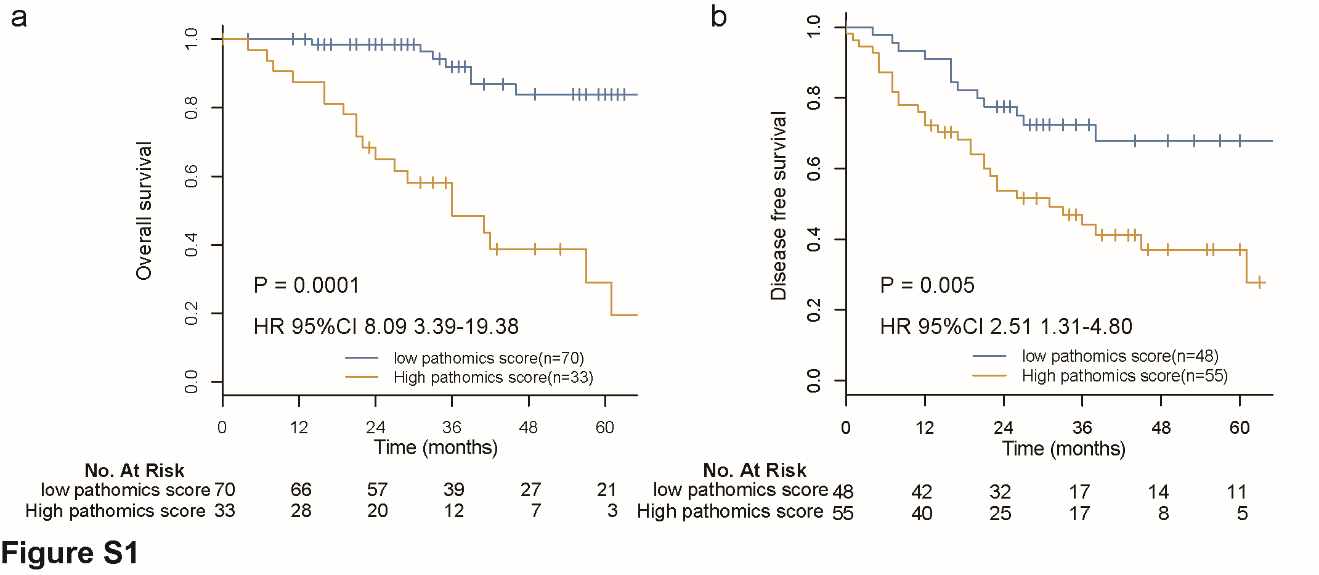
**

Additional file 2: Figure S1. Kaplan-Meier survival curves depicting OS (a) and DFS (b) according to pathomics subtypes. Significant difference in survival of patients was observed between high and low pathomics score.

**
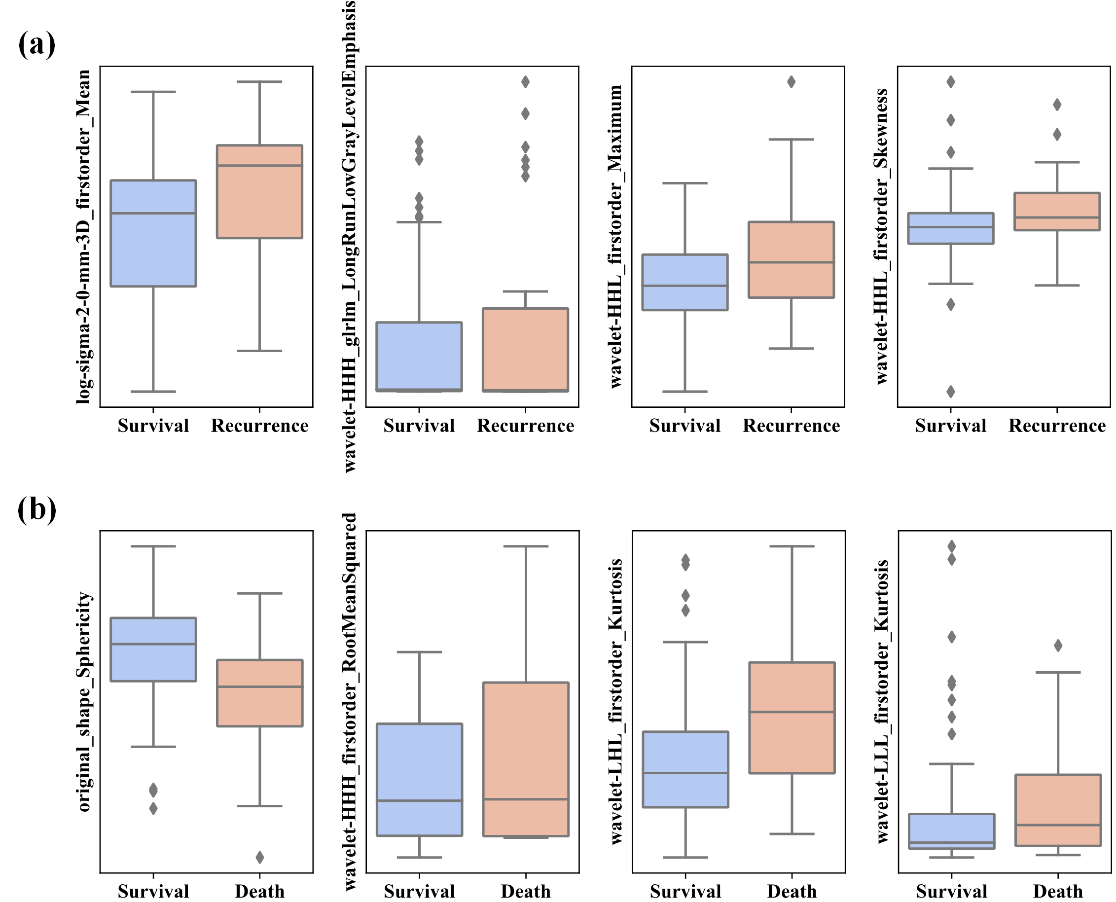
**

Additional file 2: Figure S2. Boxplots of the selected imaging features in radiomics model. (a) shows the features selected in DFS prediction model, (b) shows the features selected in OS
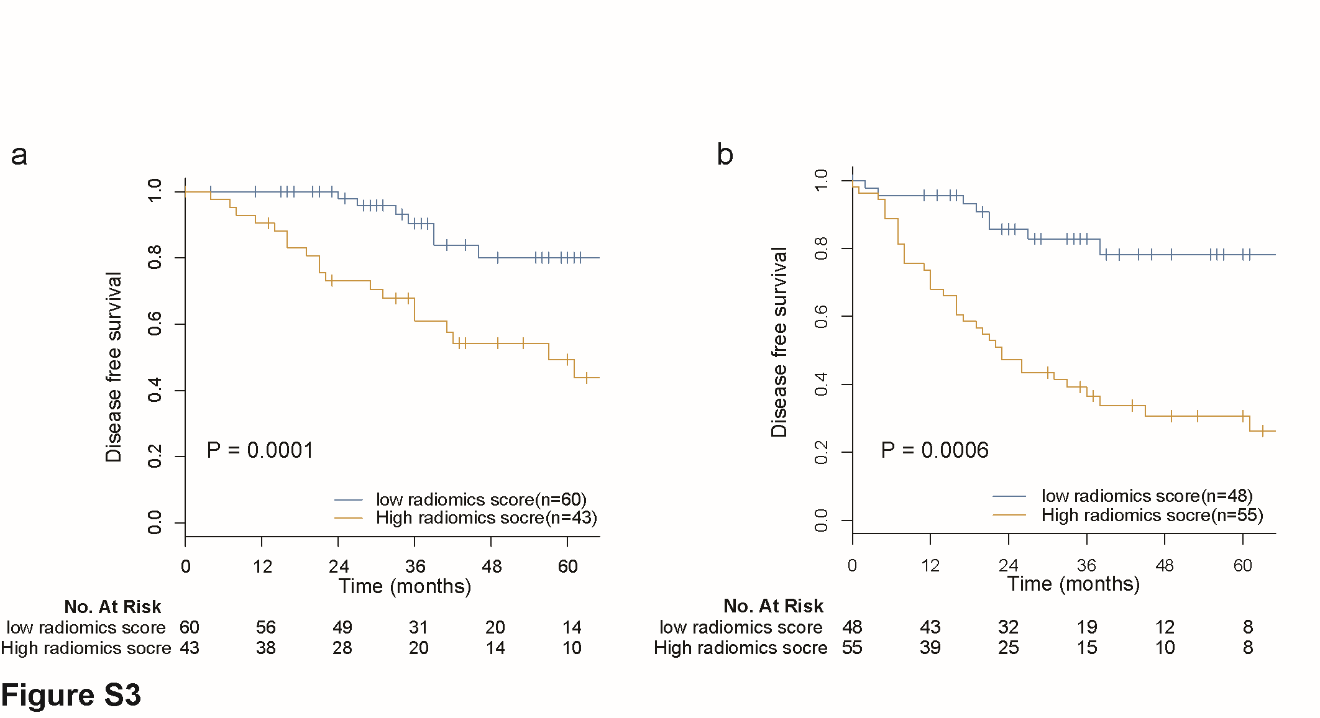
prediction mode

Additional file 2: Figure S3. Kaplan–Meier survival curve of 103 CRC patients with lung metastasis for estimating DFS (a) and OS (b) according to radiomic subtypes. The low and high radiomic score are the patients with prediction scores ≤50% and >50% respectively.

**
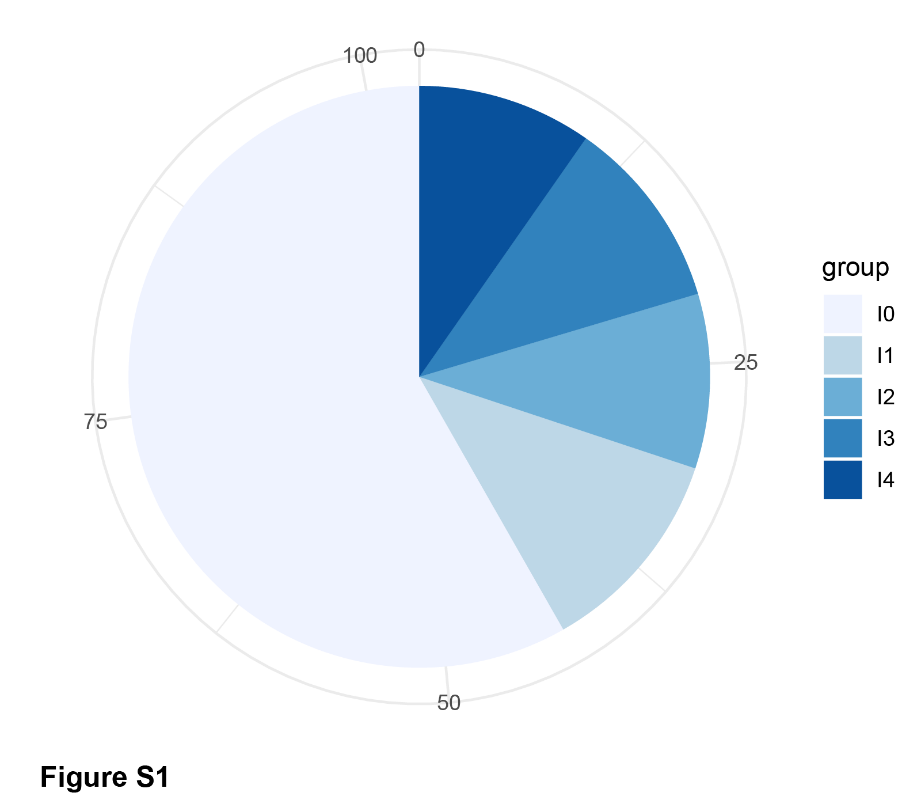
**

Additional file 2: Figure S4. Percentile distribution of Immunoscore in CRC patients with lung metastasis.

Additional file 2: **
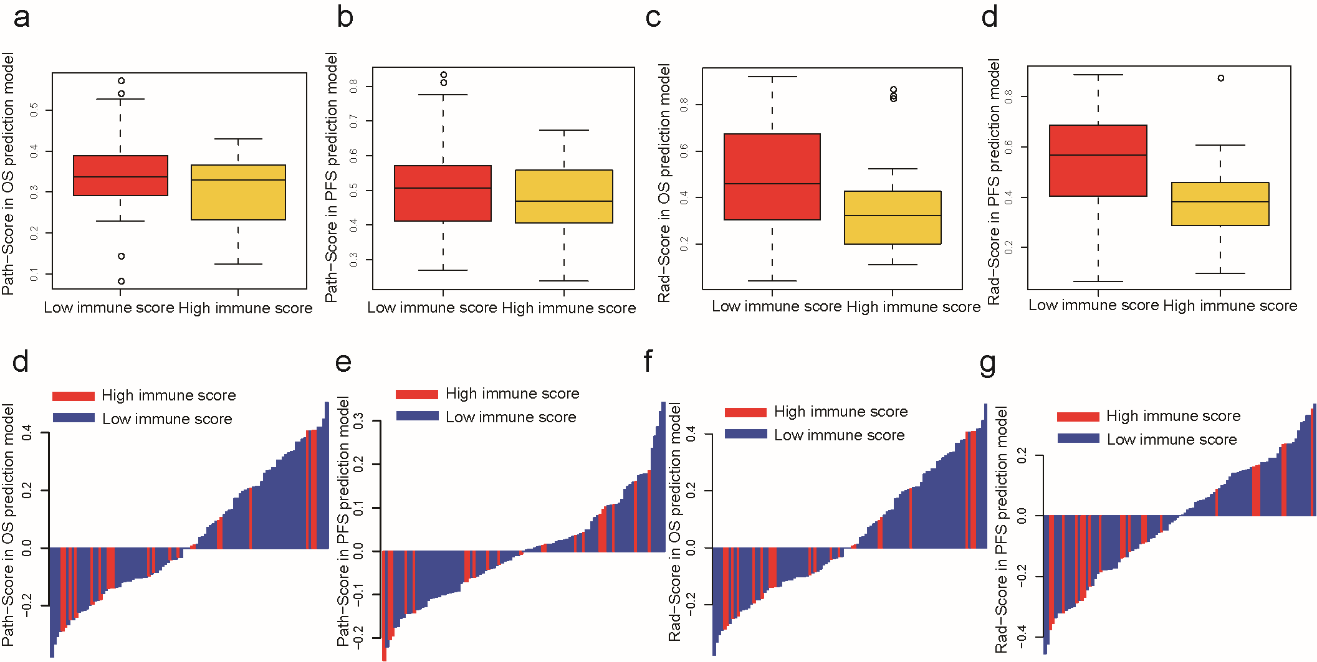
**
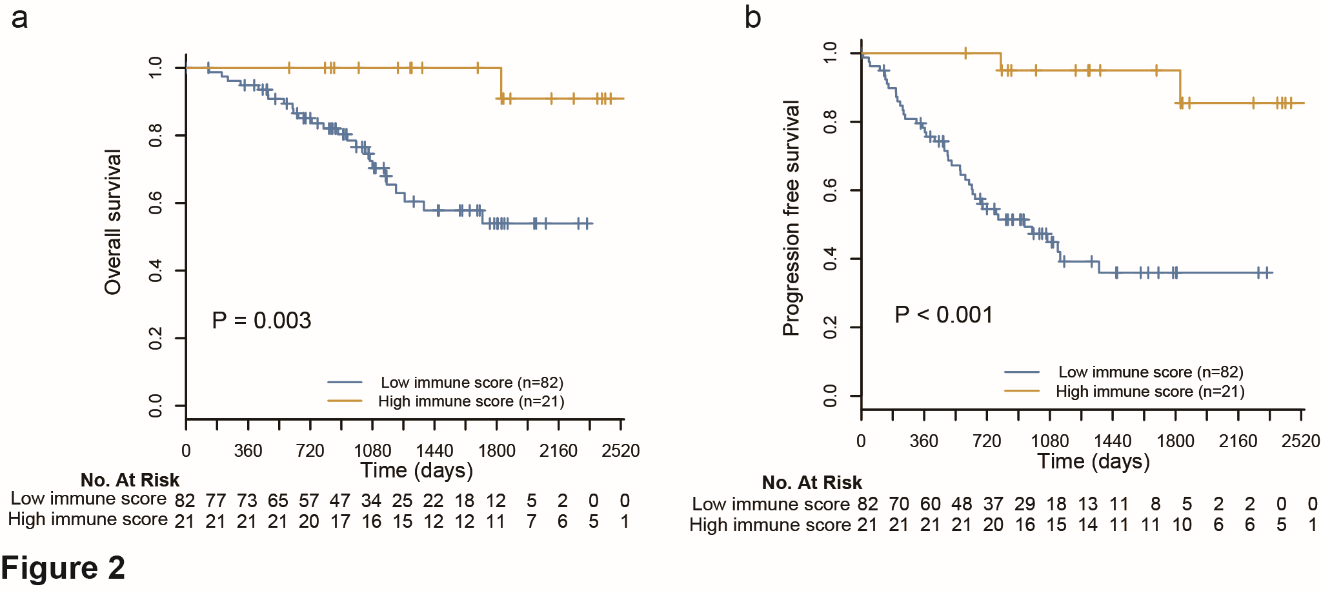
Figure S5. Kaplan-Meier survival curves depicting OS (a) and DFS (b) according to Immunoscore. Significant difference in survival of patients was observed between high and low immune score.

Additional file 2: Figure S6. Comparison of pathomics/radiomics score of OS (a, c) and DFS (b, d) prediction model between patients in low and high immune score group. Distribution of pathomics/radiomics score of OS (d, f) and DFS (e, g) prediction model and immune status.
