# Supplementary material for: Development of a novel combined nomogram model integrating deep learning-pathomics, radiomics and immunoscore to predict postoperative outcome of colorectal cancer lung metastasis patients
Source: J Hematol Oncol. 2022 Jan 24;15:11. doi: 10.1186/s13045-022-01225-3 (PMC8785554; doi:10.1186/s13045-022-01225-3)
Supplement: Supplementary file 3 — Additional file 3 . Supplementary Tables. Table S1. Clinicopathological characteristics of colorectal lung metastasis patients. Table S2. Distribution of patients with high CD3/CD8 cell density (≥75th percentile) in different regions. Table S3. Multivariate Cox analysis of OS and PFS. [file 13045_2022_1225_MOESM3_ESM.docx]

**Additional file 3: Tables**

| Additional file 3: Table S1. Clinicopathological characteristics of colorectal lung metastasis patients. | | |
| --- | --- | --- |
|  | N. | % |
| Age |  |  |
| <60 | 64 | 62.14 |
| ≥60 | 39 | 37.86 |
| Sex |  |  |
| Male | 66 | 64.08 |
| Female | 37 | 35.92 |
| Surgical approach | |  |
| Sublobar resection | 69 | 66.99 |
| Lobectomy | 34 | 33.01 |
| Number of pulmonary metastases | |  |
| 1 | 85 | 85.44 |
| >1 | 18 | 14.56 |
| Distribution |  |  |
| Unilateral | 99 | 96.12 |
| Bilateral | 4 | 3.88 |
| Hilar/mediastinal LN metastasis |  |  |
| No | 97 | 94.17 |
| Yes | 6 | 5.83 |
| Histology |  |  |
| Adenocarcinoma | 99 | 96.12 |
| Mucinous | 4 | 3.88 |
| Primary site |  |  |
| Colon | 22 | 21.36 |
| Rectum | 81 | 78.64 |
| T stage |  |  |
| T1 | 4 | 3.88 |
| T2 | 21 | 20.39 |
| T3 | 30 | 29.13 |
| T4 | 48 | 46.60 |
| N stage |  |  |
| N0 | 45 | 43.69 |
| N1 | 32 | 31.07 |
| N2 | 26 | 25.24 |
| Preoperative CEA level |  |  |
| Normal | 54 | 52.43 |
| Abnormal | 49 | 47.57 |
| Preopetative CA19-9 level |  |  |
| Normal | 89 | 86.41 |
| Abnormal | 14 | 13.59 |
| Total | 103 | 100.00 |

| Additional file 3: Table S2. Distribution of patients with high CD3/CD8 cell density (≥75th percentile) in different regions | | | | | | | | |
| --- | --- | --- | --- | --- | --- | --- | --- | --- |
|  | Center | | | | Invasive margin | | | |
|  | CD3 |  | CD8 |  | CD3 |  | CD8 |  |
|  | Low | High | Low | High | Low | High | Low | High |
| I0 | 60 | 0 | 60 | 0 | 60 | 0 | 60 | 0 |
| I1 | 9 | 3 | 9 | 3 | 8 | 4 | 10 | 2 |
| I2 | 4 | 6 | 6 | 4 | 7 | 3 | 4 | 6 |
| I3 | 4 | 7 | 2 | 9 | 2 | 9 | 3 | 8 |
| I4 | 0 | 10 | 0 | 10 | 0 | 10 | 0 | 10 |
| Total | 77 | 26 | 77 | 26 | 77 | 26 | 77 | 26 |

| Additional file 3: Table S3. Multivariate Cox analysis of OS and PFS . | | |  |  |  |  |  |  |
| --- | --- | --- | --- | --- | --- | --- | --- | --- |
|  | OS | | | | PFS | | | |
|  | HR | lower 95%CI | upper 95%CI | P | HR | lower 95%CI | upper 95%CI | P |
| Age |  |  |  | 0.558 |  |  |  | 0.490 |
| <60 | 1.00 |  |  |  | 1.00 |  |  |  |
| ≥60 | .717 | .236 | 2.181 |  | .754 | .338 | 1.681 |  |
| Sex |  |  |  | 0.042 |  |  |  | 0.023 |
| Female | 1.00 |  |  |  | 1.00 |  |  |  |
| Male | .360 | .135 | .964 |  | .433 | .208 | .901 |  |
| Surgical approach |  |  |  | 0.279 |  |  |  | 0.737 |
| Sublobar resection | 1.00 |  |  |  | 1.00 |  |  |  |
| Lobectomy | 1.864 | .604 | 5.752 |  | .882 | .425 | 1.831 |  |
| Number of pulmonary metastases | |  |  | 0.695 |  |  |  | 0.370 |
| 1 | 1 |  |  |  | 1 |  |  |  |
| >1 | .135 | .013 | 1.415 |  | .882 | .425 | 1.831 |  |
| Distribution |  |  |  | 0.103 |  |  |  | 0.789 |
| Unilateral | 1 |  |  |  | 1 |  |  |  |
| Bilateral | 8.961 | .642 | 125.073 |  | .832 | .217 | 3.188 |  |
| Hilar/mediastinal LN metastasis |  |  |  | 0.124 |  |  |  | 0.854 |
| No | 1 |  |  |  | 1 |  |  |  |
| Yes | 3.895 | .690 | 21.983 |  | .882 | .232 | 3.356 |  |
| Histology |  |  |  | 0.998 |  |  |  | 0.676 |
| Adenocarcinoma | 1 |  |  |  | 1 |  |  |  |
| Mucinous | 0.005 | 0.001 | - |  | .681 | .112 | 4.135 |  |
| Primary site |  |  |  | 0.376 |  |  |  | **0.003** |
| Rectum | 1.00 |  |  |  | 1.00 |  |  |  |
| Colon | 1.862 | .470 | 7.379 |  | 4.351 | 1.670 | 11.335 |  |
| T stage |  |  |  | 0.053 |  |  |  | 0.189 |
| T2 | 1 |  |  |  | 1 |  |  |  |
| T3 | 6.200 | 1.416 | 27.149 |  | 2.255 | .849 | 5.990 |  |
| T4 | 2.009 | .493 | 8.191 |  | 1.767 | .687 | 4.540 |  |
| N stage |  |  |  | 0.281 |  |  |  | 0.159 |
| N0 | 1.00 |  |  |  | 1.00 |  |  |  |
| N1 | 1.862 | .470 | 7.379 |  | .482 | .169 | 1.374 |  |
| N2 | .320 | .077 | 1.334 |  | 1.353 | .556 | 3.295 |  |
| Preoperative CEA level |  |  |  | 0.708 |  |  |  | 0.926 |
| Normal | 1.00 |  |  |  | 1.00 |  |  |  |
| Abnormal | 1.229 | .417 | 3.621 |  | .961 | .419 | 2.203 |  |
| Preopetative CA19-9 level |  |  |  | 0.946 |  |  |  | 0.101 |
| Normal | 1.00 |  |  |  | 1.00 |  |  |  |
| Abnormal | 1.054 | .234 | 4.749 |  | .364 | .109 | 1.218 |  |
| Pathomics siganture | 58.805 | 2.904 | 119.079 | 0.019 | 65.198 | 4.687 | 906.841 | 0.002 |
| Radiomics signature | 47.503 | 2.739 | 823.911 | 0.008 | 61.790 | 5.973 | 639.177 | <0.001 |
| Immunoscore |  |  |  | 0.033 |  |  |  | 0.002 |
| Low immune score | 1.00 |  |  |  | 1.00 |  |  |  |
| High immune score | .065 | .005 | .802 |  | .074 | .014 | .379 |  |
|  |  |  |  |  |  |  |  |  |

LN: Lymph node
